# Superantigen Recognition and Interactions: Functions, Mechanisms and Applications

**DOI:** 10.3389/fimmu.2021.731845

**Published:** 2021-09-20

**Authors:** Anthony M. Deacy, Samuel Ken-En Gan, Jeremy P. Derrick

**Affiliations:** ^1^School of Biological Sciences, Faculty of Biology, Medicine, and Health, University of Manchester, Manchester, United Kingdom; ^2^Antibody & Product Development Lab, Experimental Drug Development Centre – Bioinformatics Institute (EDDC-BII), Agency for Science Technology and Research (ASTAR), Singapore, Singapore; ^3^James Cook University, Singapore, Singapore

**Keywords:** superantigen, T-cell, B-cell, cytokine storm, interface, antibody purification

## Abstract

Superantigens are unconventional antigens which recognise immune receptors outside their usual recognition sites e.g. complementary determining regions (CDRs), to elicit a response within the target cell. T-cell superantigens crosslink T-cell receptors and MHC Class II molecules on antigen-presenting cells, leading to lymphocyte recruitment, induction of cytokine storms and T-cell anergy or apoptosis among many other effects. B-cell superantigens, on the other hand, bind immunoglobulins on B-cells, affecting opsonisation, IgG-mediated phagocytosis, and driving apoptosis. Here, through a review of the structural basis for recognition of immune receptors by superantigens, we show that their binding interfaces share specific physicochemical characteristics when compared with other protein-protein interaction complexes. Given that antibody-binding superantigens have been exploited extensively in industrial antibody purification, these observations could facilitate further protein engineering to optimize the use of superantigens in this and other areas of biotechnology.

## Introduction

Superantigens are unconventional antigens in the sense that they elicit a response by binding outside the complementary determining regions (CDRs) of their target immune receptor macromolecules (antibodies or T-cell receptors). At their initial description in 1989, superantigens were originally defined as proteins that hyper-stimulate T-cells *via* the crosslinking of T-cell receptors (TCRs) and MHC Class II molecules (1, 2). This definition required extension following the discovery of B-cell superantigens. B-cell superantigens can hyper-stimulate a large population of B-cells without necessarily having the ability to crosslink TCRs with MHC Class II receptors; they therefore have a different mechanism and specificity compared to T-cell superantigens (3). B-cell superantigens are commonly known to (i) stimulate a high proportion of B-cells, and (ii) bind outside of the CDRs (4). An extended definition of the term ‘superantigen’ was suggested to incorporate both functions, as a molecule which has antigen-receptor mediated interactions with over 5% of the lymphocyte pool (5). This functional definition is therefore based on the hyper-activity of the target receptor upon exposure, and we will use the term in this context here.

Here we review the current understanding of superantigens, how they directly interact with immune receptors of T and B-cells, what common features may be identified in recognition interfaces and how such insights could be adapted to facilitate further protein engineering of these versatile macromolecules for therapeutic, diagnostic, and biotechnological applications.

## T-Cell Superantigens

T-cell superantigens are typically microbial proteins. They were first identified from observation of the hyper-stimulation of T-cells by *Staphylococcal Enterotoxin B* (SEB). This phenomenon was caused by the crosslinking of T-cell receptors (TCRs) Vβ with MHC class II α_1_ on antigen presenting cells (APC) by SEB (1, 2). By crosslinking MHC Class II to TCR, small amounts of superantigens can stimulate extensive T-cell proliferation. In a normal adaptive immune response, only around 0.0001% of T-cells are activated. In contrast, superantigen exposure can activate up to 30% of the T-cell pool, leading to severe pathologies following infection (6, 7).

**Figure 1:**
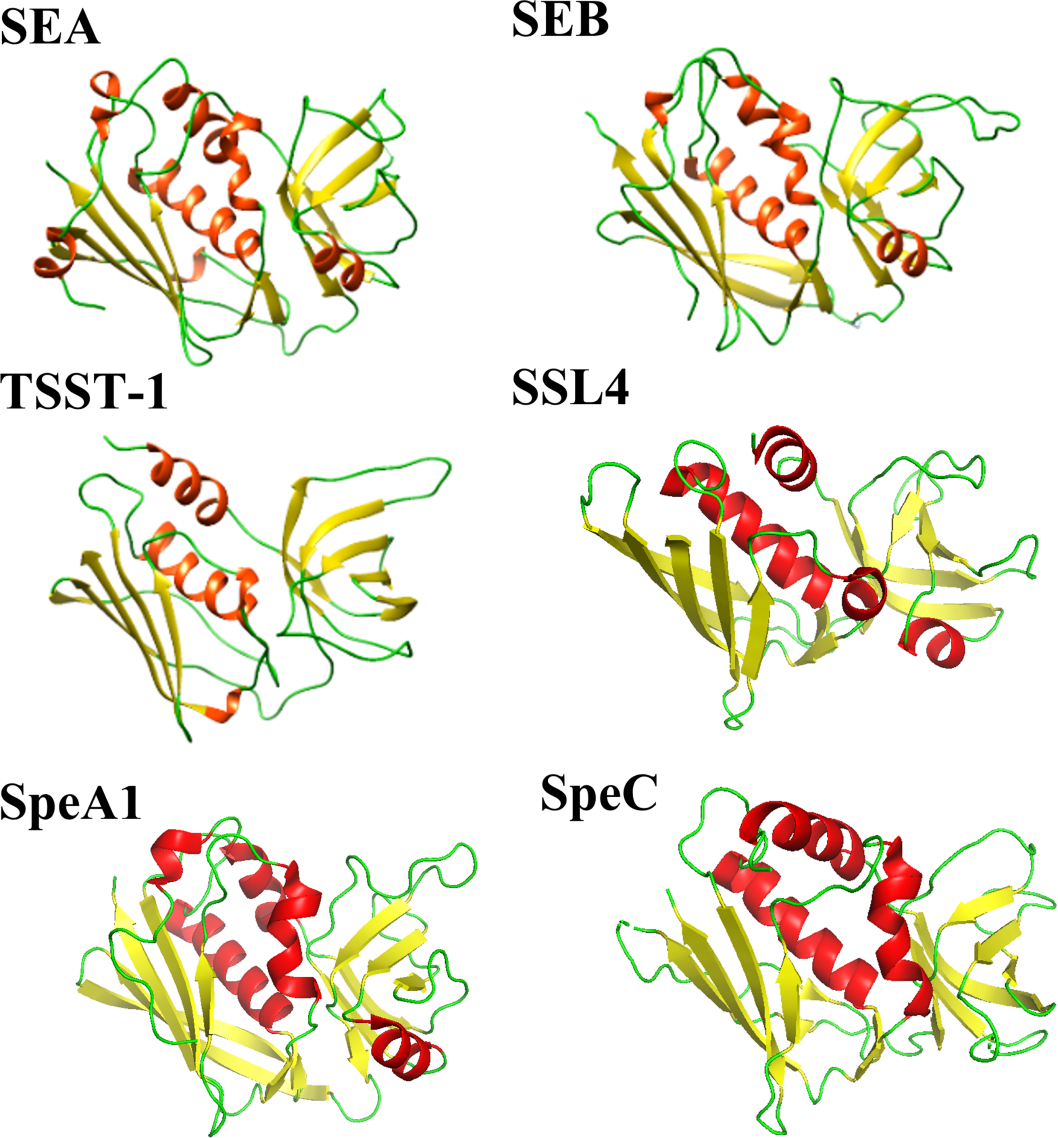
Comparison of selected *Staphylococcal aureus* and *Streptococcus pyogenes* enterotoxin and enterotoxin-like structures. *Staphylococcal aureus* enterotoxins include SEA (PDB code: 1ESF), SEB (PDB code: 1SE4) and TSST-1 (PDB code: 2QIL). The *Staphylococcal aureus* enterotoxin-like protein shown is SSL4 (PDB code: 4DXF). Two *Streptococcal pyogenes* enterotoxins are displayed: SpeA1 (PDB code: 1UUP) and SpeC (PDB code: 1KTK). The structures are shown as a ribbon plot with α-helices, β-strands and loops coloured in red, yellow, and green, respectively.

Enterotoxins produced by *Staphylococcus aureus* and *Streptococcus pyogenes* form a common family of T-cell superantigens. These enterotoxins are small (20-28 kDa), two domain proteins which are diverse in sequence (15-90%) (8). Despite this variation, enterotoxins and enterotoxin-like proteins from both *Staphylococcus aureus* and *Streptococcus pyogenes* are structurally similar (**Figure 1**), possessing a conserved Greek key motif at the N-terminus known as an oligonucleotide (OB)-fold (9). The C-terminus consists of a conserved β-domain capped by an α-helix (9) with the two β-folds separated by a cluster of α-helices. Due to their structural similarity, it has been suggested by others that the enterotoxins from *Staphylococcus aureus* and *Streptococcus pyogenes* shared a common ancestor (8).

Enterotoxins are thermostable, can withstand extreme pH and are resistant to degradation by proteolytic enzymes such as pepsin and trypsin (10, 11). Some can retain activity after the cooking and digestive process to cause food poisoning (12): nearly 25% of food poisoning cases in the USA are attributed to *Staphylococcal* enterotoxins (13). In addition, T-cell superantigens also contribute to the development of systemic inflammatory response syndrome (SIRS) known as sepsis (14), toxic shock syndrome (15, 16), scarlet fever (17) and atopic dermatitis (18).

## Cellular Responses to T-Cell Superantigens

Observations of the cellular responses of T-cells to superantigens are inconsistent, depending on the type and maturity of the T-cell populations studied. T-cell superantigens can cause immature CD4^+^ and CD8^+^ T-cells to become depleted. Mature CD4^+^ and CD8^+^ T-cells on the other hand, proliferate and produce a cytokine storm (19–25) driving mature T-cells into a state of anergy (26). TCR activation upregulates Fyn signalling, preventing the protein tyrosine kinase ZAP-70 from associating with TCRs *via* CD3, thus inhibiting TCR signalling (27). The depletion of immature T-cells and anergy of mature T-cells would potentially allow a pathogen to evade the innate immune response, increasing pathogen survivability.

**Figure 2:**
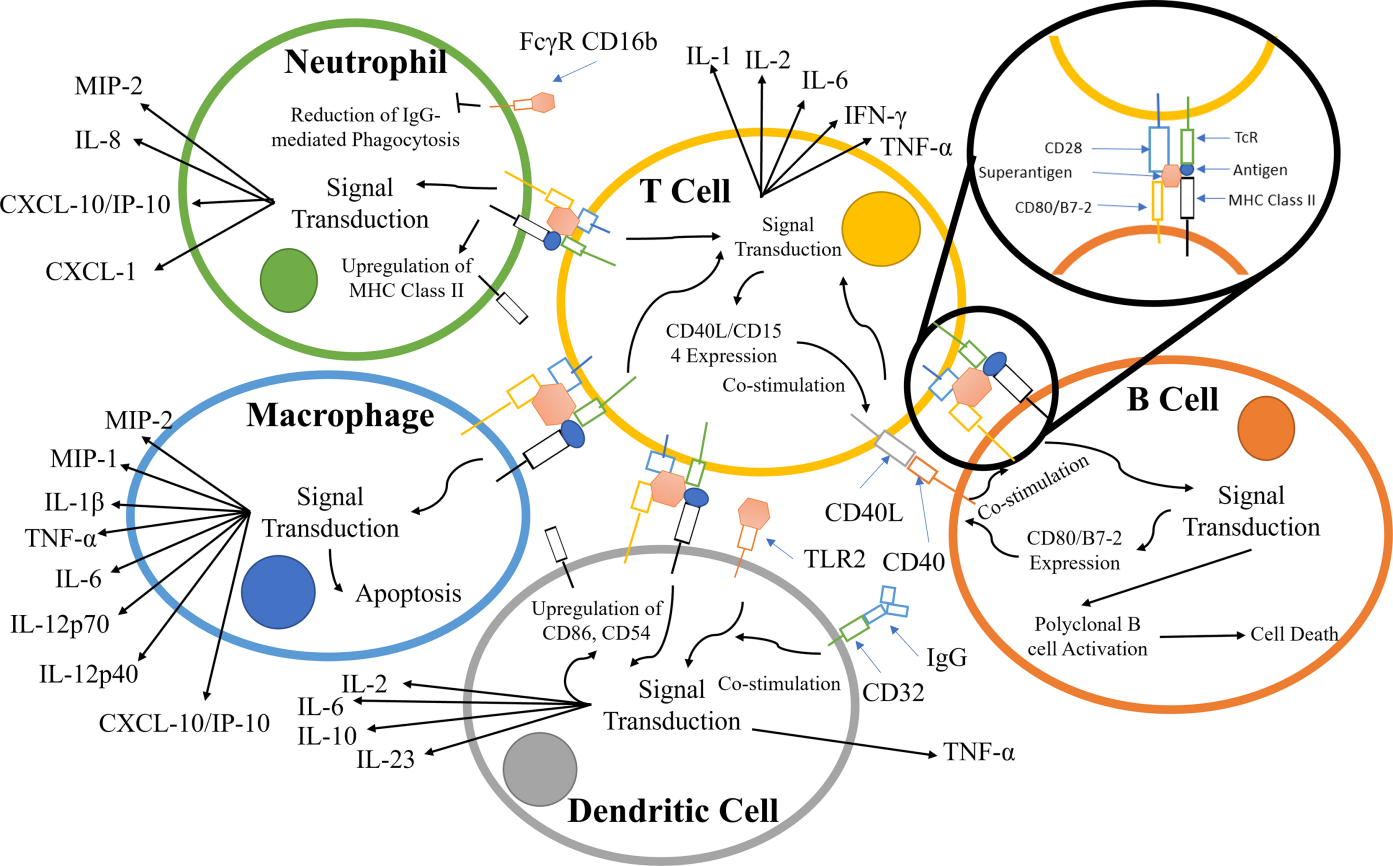
Principal components involved in the superantigen activation of T-cells, B-cells, macrophages, and neutrophils. The interactions displayed are based on material from references (28–49). The responses contribute to and escalate the hyper-activation of T-cells and subsequent cytokine storm.

TCR binding to the MHC class II receptors on APCs results in a variety of responses that is dependent on the APC type; the principal pathways and components are summarized in **Figure 2**. During infection, neutrophils are recruited along with other effector cells through the release of cytokines (e.g. IFN-γ, IL-17, IL-12) and CXC chemokines produced primarily by CD4^+^ T-cells (28, 50–52). Counterintuitively, the recruitment of leukocytes increases the survivability of *Staphylococcus aureus*, due to the hyper-stimulation of T-cells, eventually leading to T-cell anergy and cell death. *S. aureus* is known to survive within neutrophils and macrophages in abscesses (29, 30).

Alongside TCR/MHC Class II activation, signalling pathways are co-stimulated by crosslinking CD28 on the T-cell with CD80/B7-2 on APCs (53–56). T-cell superantigens can also crosslink the α-subunit of laminin, LAMA2, with G-protein coupled receptor (GPCR), resulting in T-cell stimulation (57–59) (**Figure 2**).

## MHC Class II Binding

**Figure 3:**
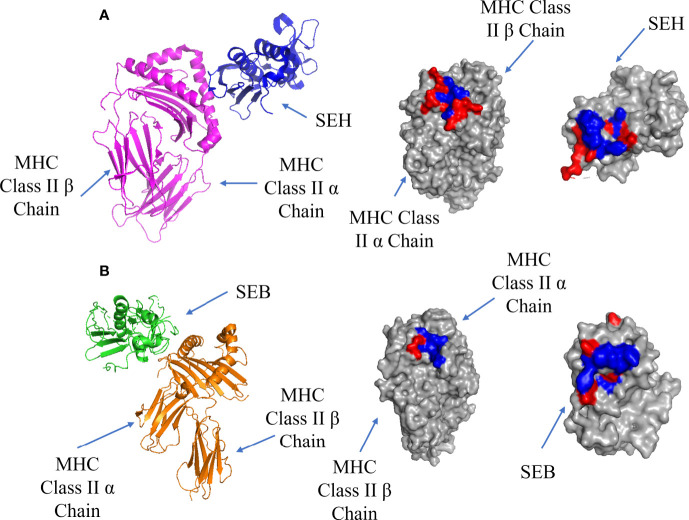
Binding of SEH and SEB superantigens to -MHC Class II. **(A)** Left panel: ribbon plot of SEH (blue) bound to the high affinity site on MHC Class II α Chain (purple) (PDB code: 2XN9). Middle Panel: MHC Class II showing polar (red) and hydrophobic residues (blue). Right Panel: SEH. **(B)**. Left panel: ribbon plot of SEB (green) bound to the low affinity site on MHC Class II β Chain (orange) (PDB code: 1SEB). Middle panel: MHC Class II β Chain. Right panel: SEB.

T-cell superantigens first bind to MHC Class II receptors and accumulate on the surface of the APC before binding to the TCR (9). There are two possible binding sites on MHC Class II: a Zn-dependent high affinity site (K_d_ = 10^-7^ - 10^-8^ M) located on MHC Class II β chain, and a low affinity site (K_d_ = ~10^-5^ M) located on MHC Class II α chain (60). Most superantigens bind *via* the Zn-dependent binding site, forming a complex which is stable for more than 40 hours (61). The high affinity interface between SEH and MHC Class II α chain is shown in **Figure 3A**, showing a hydrophobic pocket surrounded by polar residues. In addition to H-bonds and salt bridges, a Zn ion contributes to the high binding affinity by stabilizing the complex through crosslinking H81 on the MHC Class II β-strand and H206, N208 on the β-strand 12 on SEH (62). This stabilization allows for the formation of 4 extra H-bonds due to the proximity of the chains where the removal of the Zn ion results in a decrease in binding for SEA, SED, SEE and SEH (63).

The low affinity binding site is exemplified by a structure containing the enterotoxin SEB, which forms a complex with MHC class II (61): the low affinity interface is shown in **Figure 3B**. In addition to cross-interface bonds, there is a hydrophobic patch on SEB comprised of F44, L45 and F47 which inserts into a hydrophobic pocket on MHC Class II α chain.

The enterotoxin SEA can also bind to the low and high affinity sites to crosslink two MHC Class II molecules (61, 64, 65). *Staphylococcal Enterotoxin H* (SEH) was shown to bind the Zn-dependent high affinity site on MHC class II (62), as well as to TCR Vα instead of Vβ (66, 67). A list of T-cell superantigens and their site specificities has been previously summarized by Proft and Fraser (9). T-cell superantigen selectivity for the α or β chains of the MHC Class II complex is dependent on the presence of the Zn atom at the C-terminal β domain. Its absence leads to the binding of the α-chain of MHC Class II *via* a hydrophobic ridge on the N-terminal OB-domain (9).

## T-Cell Receptor Binding

**Figure 4:**
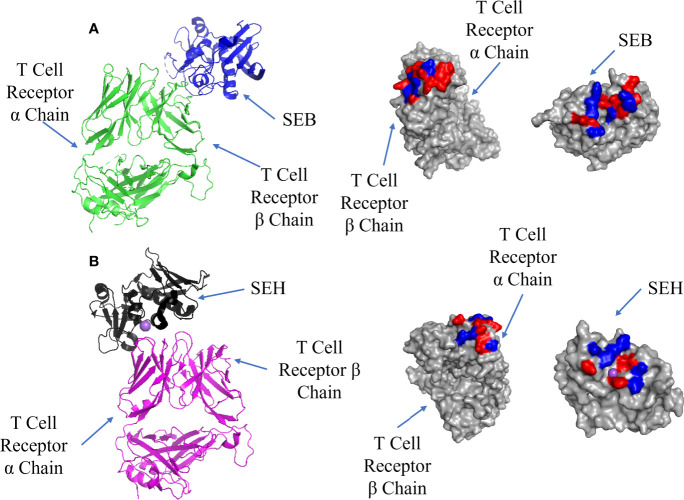
Binding of SEB and SEH superantigens to -TCRs. **(A)** Left panel: ribbon plot of SEB (blue) bound to TCR (green) (PDB code: 4C56). Middle panel: TCR showing polar (red) and hydrophobic residues (blue). Right panel: SEB. **(B)** Left panel: ribbon plot of SEH (black) bound to TCR (purple) (PDB code: 2XN9). Middle panel: TCR. Right panel: SEH.

Superantigens bind to the TCR after adhesion to MHC Class II; there are also two sites on the TCRs in all superantigen complexes studied to date. Some T-cell superantigens bind to the α chain [SEH (68)], although most recognize the β chain. Unlike complexes with MHC Class II, both TCR interfaces bind T-cell superantigens at low affinity (K_d_ = 10^-4^ – 10^-6^ M) (68, 69) and yet both are capable of mediating activation of a cytokine storm (8, 70–73). SEB binding to the TCR β chain is shown in **Figure 4A** where the interface is located at the TCR binding cleft between the N-terminal β-barrel and the second α-helix. It is characterised by several cross-interface bonds, with N23 playing a crucial role, and a nearby hydrophobic patch formed of V26, Y79 and Y80 on SEH packing against the CDR2 loop of TCR Vβ (74).

SEH binding to the TCR α chain forms an interface comprising hydrophobic and hydrophilic patches, with a notable hydrophilic patch surrounding a Na ion (**Figure 4B**). Comparing this to the SEB-TCR β chain interface, there are 7 fewer H-bonds and 2 fewer salt bridges, although the binding affinities are similar (K_d_ = 10^-4^ – 10^-6^ M) (68). The lack of contacts between SEH and the TCR α chain may be bolstered by the presence of the Na ion. N16 found on the second α-helix and the hydrophobic patch (Y79 and Y80) on SEH are well conserved among T-cell superantigens whether they bind to the TCR α or β chains (67). The mutation N23A (equivalent to N16 in SEH) in SEC2 caused the loss of mitogenic activity (75) and the same mutation in SEB resulted in poorer proliferation of T-cells (76).

## Binding of B-Cell Superantigens

B-cell superantigens bind immunoglobulins outside the CDRs; proteins which would fit this definition of a superantigen were first described in the early 1990s (3). Binding to the Fab fragment drives B-cells into apoptosis by hyper-activation of B-cell receptors (BCRs). Considering that 20 to 50% of B-cells have BCRs on their surfaces (77), B-cell superantigens can elicit a potent immune response. However, B-cell superantigens are better known for their ability to bind Fc and their applications as affinity resins for antibody purification.

**Figure 5:**
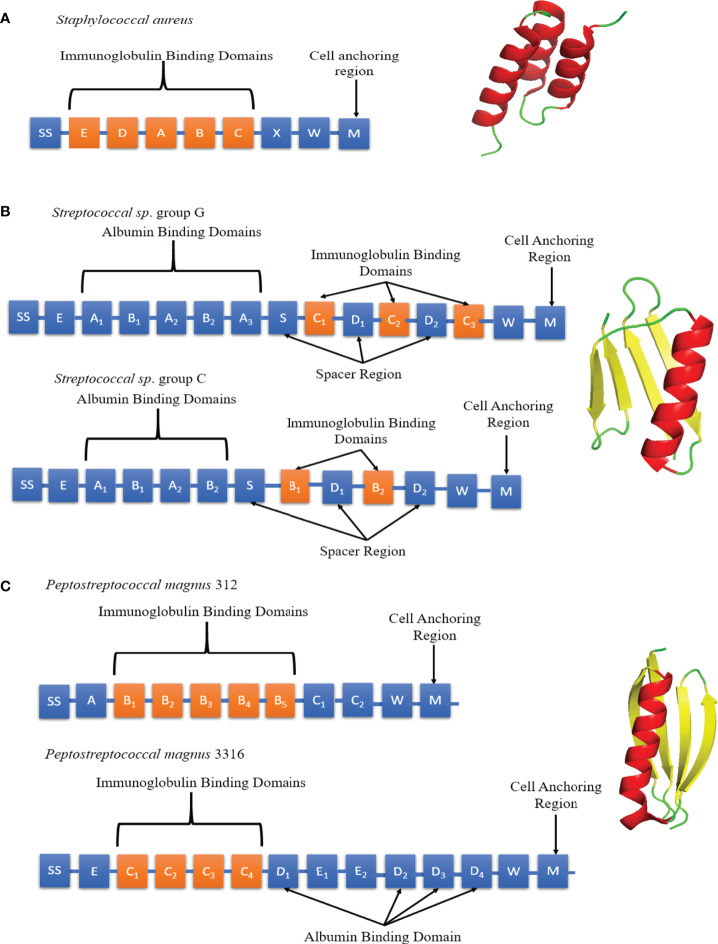
Schematic diagrams of SpA, SpG and PpL domain structures. **(A)** Left panel: Individual SpA domains including S (sorting peptide), Domains E-D-A-B-C, Region X and Region M. Right panel: SpA Domain C (PDB code: 4WWI) Each SpA immunoglobulin binding domains consists of 3 α-helices (red). **(B)** Left panel: Individual SpG domains including S (sorting peptide), Region E, Albumin Binding Domains A1-A2-A3, immunoglobulin binding domains B1-B2/C1-C2-C3 and Region W. Right panel: SpG Domain B1 (PDB code: 3GB1) Each SpG immunoglobulin binding domain consists of 1 α-helix (red) and 4 anti-parallel β-strands (yellow). **(C)** Left panel: Individual PpL domains including S (sorting peptide), Immunoglobulin Binding Domains B1-B2-B3-B4-B5/C1-C2-C3-C4, Albumin Binding Domains D1 to D4, Region W and M. Right panel: PpL Domain B1 (PDB code: 1HEZ). Each PpL immunoglobulin binding domain consists of 1 α-helix (red) and 4 anti-parallel β-strands (yellow).

*Staphylococcal* Protein A (SpA), *Streptococcal* Protein G (SpG) and *Peptostreptococcal* Protein L (PpL) are B-cell superantigens located on the bacterial cell wall (78, 79). SpA was classified as a superantigen in 1995 due to its observed effect on B-cells (4). However, SpA was first isolated in 1940 and identified in 1964 due to its Fc binding ability (78, 80). It comprises a 42 kDa protein arranged into five homologous domains (E-D-A-B-C), each forming a three α-helix bundle fold (**Figure 5A**) (81, 82). The domains are linked by conserved, flexible linkers (82). Native SpA also includes region X, a 12 x 8-residue repeat sequence which binds peptidoglycan. All 5 A-E domains can bind both Fc and Fab fragments (83). The binding affinity for specific immunoglobulins depends on the isotype and species origin. In humans, SpA binds strongly to IgG1, IgG2, IgG4 and weakly to IgA1, IgA2 and IgM. Mutations R435H and F436Y on hIgG3 have been identified as the reason SpA cannot bind human IgG3 (84). Interestingly, mutations in CDR2 from the therapeutic antibodies Herceptin and Pertuzumab were shown to contribute to binding SpA (85).

SpG was first identified in 1984 by Björck and Kronvall (86) and subsequently described as a B-cell superantigen. The sequence of SpG differs depending on the *Streptococcus* strain of origin (**Figure 5B**). SpG from group C *Streptococcus* sp. contains 2 immunoglobulin binding domains (B1-B2) whereas group G has 3 (C1-C2-C3) (87–89). Between each immunoglobulin binding domain are ‘spacers’, known as D domains. All SpG immunoglobulin-binding domains can bind both the Fc and Fab fragments (83). SpG has provided an alternative to SpA in antibody manufacturing, due it its ability to bind some antibody isotypes not recognised by SpA. It can strongly bind to all four human IgG subclasses (IgG1, IgG2, IgG3 and IgG4).

PpL was shown to induce apoptosis in B-cells by binding to the V_L_ region outside of the CDRs of BCRs, fulfilling the definition of a B-cell superantigen (90). It was first isolated in 1985 and characterised as an immunoglobulin-binding protein capable of binding to the variable light chain in 1988 (79, 91). Of the two most common strains of *Peptostreptococcus magnus*, strain 312 produces a 79 kDa, 5 domain (B1-B2-B3-B4-B5) protein whereas strain 3316 expresses a 106 kDa 4 domain (C1-C2-C3-C4) protein (**Figure 5C**) (92). PpL recognizes the light chain exclusively and cannot bind to the Fc region. This makes it highly suitable for affinity-purification of non-IgG antibodies (93, 94).

All three B-cell superantigens (SpA, SpG and PpL) share several common features; they form small, stable, multidomain structures with a ‘beads on a string’ type structure. Kim *et al*. compared antibody levels of IgG and V_H_3+ IgM in mice when infected with SpA mutants with one to 6 domains. The results showed that the optimal number of immunoglobulin binding domains to induce the largest B-cell response was 5 (95). This observation suggests that B-cell superantigens are driven by the need for multivalency of binding and the consequent improved cross-linking of BCRs. These results were corroborated by a similar study with PpL (92). Although SpG and PpL share no significant sequence homology (15%), their immunoglobulin binding domains have similar folds, forming a β-sheet packed against a single α-helix. A gene transfer event between *Streptococcus aureus* and *Peptostreptococcus magnus* has been proposed to explain a possible common evolutionary origin of SpG and PpL (96). All three B-cell superantigens also utilise regions W and M for crossing the cell membrane, featuring the common Gram-positive protein anchoring motif LPXTG (97, 98). SpG and PpL also contain albumin binding domains (99, 100), which are absent in SpA.

## Cellular Responses to B-Cell Superantigens

**Figure 6:**
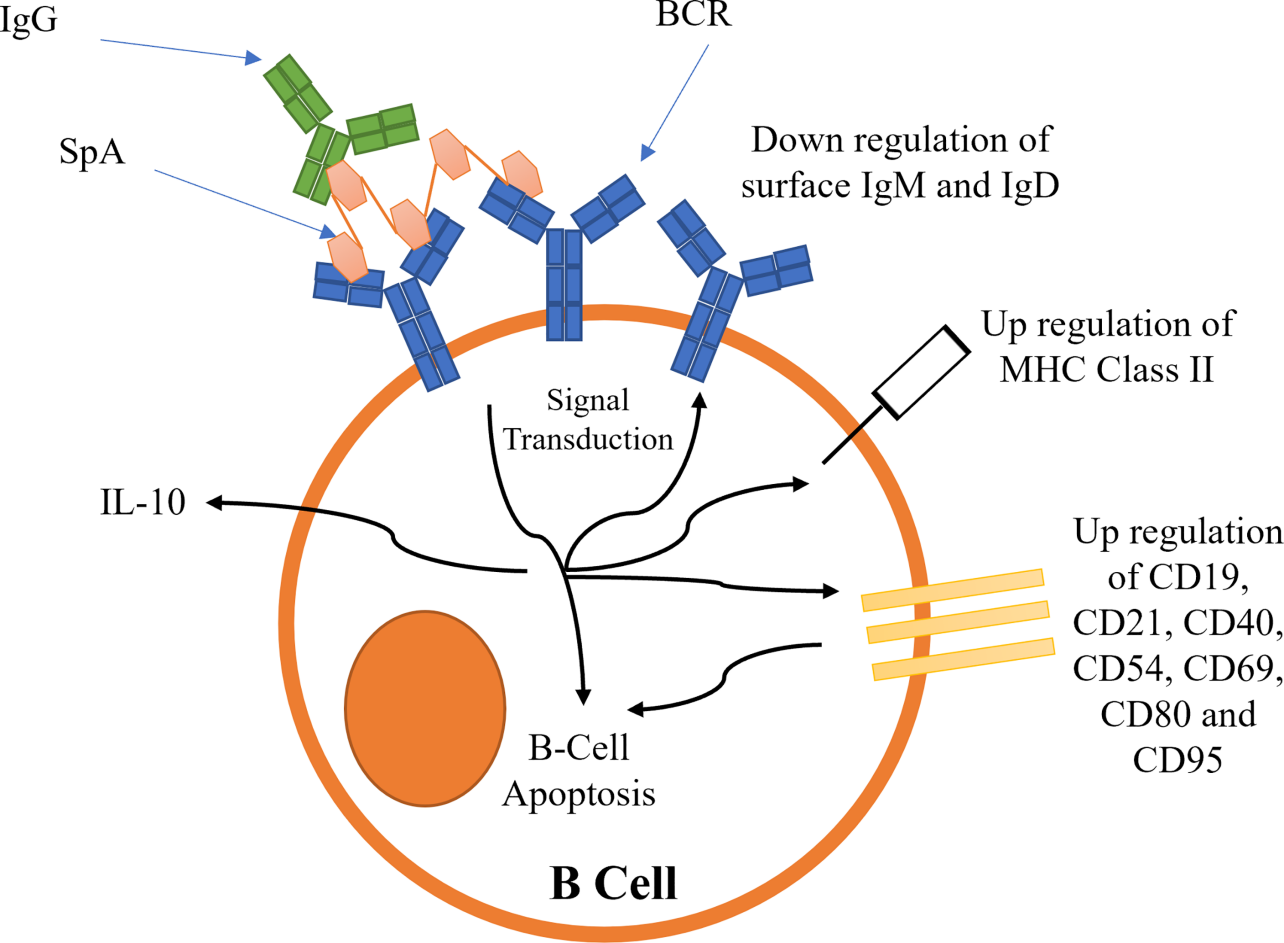
Proposed mechanisms for the activation of B-cell receptors by SpA. Activation leads to B-cell capping and B-cell apoptosis.

B-cell superantigens cross-link BCRs to activate BCR dependent signalling (101, 102). This initial signal transduction leads to the downregulation of BCRs, and an upregulation of several cluster of differentiation (CD) receptors (102), resulting in B-cell capping (summarized schematically in **Figure 6**). MHC Class II is also upregulated (102). The upregulation of these receptors leads to the activation of pro-apoptotic signals, such as Caspase 3, causing mitochondrial permeabilization and apoptosis (5, 101, 102). Recently it has been shown that SpA B-cell superantigen activity is dependent on the presence of the LPXTG anchoring motif as well as the ‘LysM domain’ between region X and the LPXTG motif (103). These observations imply SpA must be bound to peptidoglycan to cause B-cell stimulation.

The precise functional role of microbial B-cell superantigens binding to Fc is obscure, although it has been shown recently that soluble IgG is a requirement for the successful activation of BCRs by SpA (104). The efficiency of BCR activation was dependent on the strength of Fc binding to each IgG subclass (104). The binding of SpA-IgG complex to BCR is predicted to increase the functional valency of the complex (104). SpA-IgG is thought to form a ‘lattice’ structure around the B-cells by crosslinking BCR Fab with IgG Fc and other BCR Fab regions promoting a sustained stimulation.

Other potential functions of the B-cell superantigen-immunoglobulin interaction are the blocking of immunoglobulin effector functions, opsonization and immunoglobulin-mediated phagocytosis, antibody-dependent cell mediated cytotoxicity (ADCC) and complement-dependent cytotoxicity (CDC) (105–107). Expression of B-cell superantigens ultimately leads to B-cell depletion and evasion of the immune system: in this sense, they can be considered as virulence factors (108–111).

SpA, SpG and PpL bind to BCRs at different sites on the Fab fragment, although the activation results in similar cellular responses. SpG binds to the C_H_1 domain (112), implying isotype dependent binding, whereas SpA binds to the V_H_3 family only. A comparison of the conservation of key residues between the seven V_H_ families shows that, although many residues are conserved, there are several which are key and which, when mutated, result in the loss of binding for SpA (113), including in the V_H_-CDR2 (85). PpL domains only bind the κ light chain V_L_ region and therefore lacks the ability to bind λ chains. The binding affinity of PpL differs between the families of κ light chain, specifically to FW1: it can bind to human Vκ I, III and IV, but not II (114, 115).

Several T-cell superantigens have the ability to bind BCRs, although generally in a weak and non-specific manner (4), and without a B-cell response. Exceptions have been noted, for example, SEA increased the survival of V_H_3 B-cells (116). SED has also been shown to increase survival of V_H_4 B-cells (117). However, the *in vivo* response is yet to be determined.

Recent research suggests that B-cell superantigens also enhance immune defences (118). Two superantigens have been identified from the commensal bacteria *Lachnospiraceae sp*: Immunoglobulin Binding Proteins A (IbpA) and B (IbpB). Both were observed to activate BCRs by binding V_H_3 leading to the increased secretion of IgA, although this was only shown *in vitro*.

## B-Cell Superantigen-Fab Complex Interfaces

**Figure 7:**
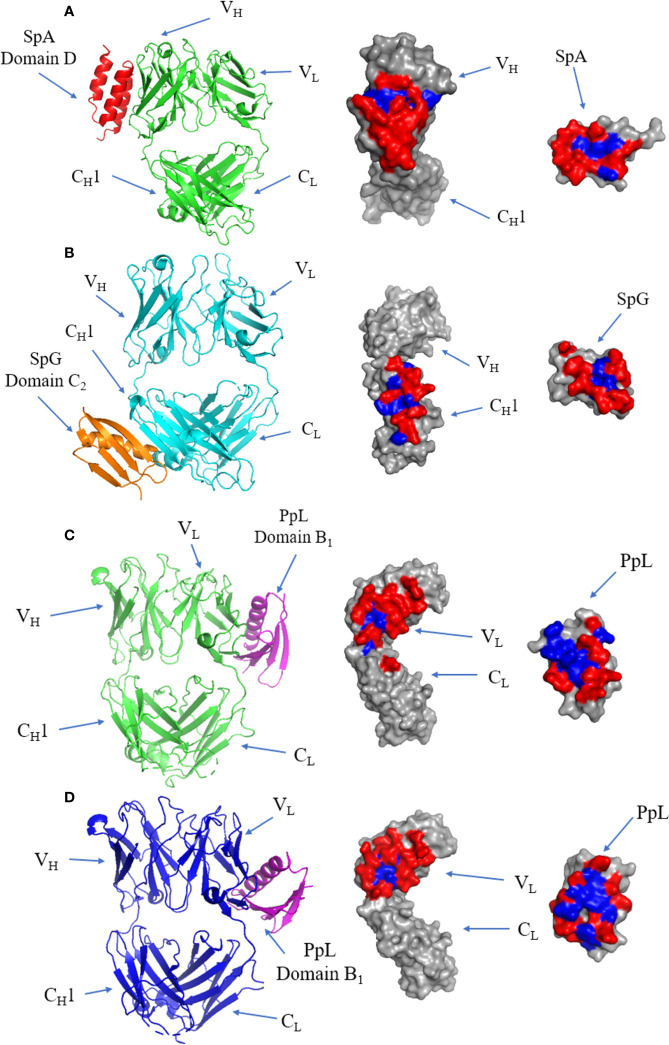
Binding of SpA, SpG and PpL to antibody Fab fragments: **(A)** Left panel: ribbon plot of SpA (Red) bound to V_H_3 domain of IgM Fab (Green) (PDB code: 1DEE). Middle panel: IgM Fab showing polar (red) and hydrophobic residues (blue). Right panel: SpA. **(B)** Left panel: ribbon plot of SpG (Orange) bound to C_H_1 domain of IgG Fab (Cyan) (PDB code: 1QKZ). Middle panel: IgG Fab. Right panel: SpG. **(C)** Left panel: ribbon plot of PpL Domain B_1_ (Purple) interface 1 bound to IgM Fab (Green) at the V_L_ domain (PDB code: 1HEZ). Middle panel: IgM Fab. Left panel: PpL. **(D)** Left panel: ribbon plot of PpL Domain B_1_ (Purple) interface 2 bound to IgM Fab (Blue) at the V_L_ domain (PDB code: 1HEZ). Middle panel: IgM Fab. Right panel: PpL.

The crystal structure of the SpA and IgM Fab complex is illustrated in **Figure 7A**, showing that the interface occurs at the V_H_ domain (involving residues from β-strands B to E) of the Fab fragment and α-helices 2 and 3 of SpA (113). The interface is dominated by polar residues with three negatively charged residues from SpA and two positively charged residues from Fab forming electrostatic interactions (113). All SpA domains can bind to the Fab fragment (119), and each domain varies in its affinity towards V_H_3. The interacting residues form a predominantly hydrophilic interface forming several cross-interface bonds.

SpG domain C2 co-crystalized with IgG Fab (**Figure 7B**): the interface forms an antiparallel alignment between the last β-strand of the C_H_1 domain and the second β-strand of SpG (120). The antiparallel complex also results in interactions occurring between first β-strand of C_H_1 and the C-terminal end of the α-helix of SpG (121). The interface is formed by mostly hydrophilic residues flanking a small hydrophobic patch.

The first of two binding sites of PpL to IgM Fab is shown in (**Figure 7C**). The majority of the interface occurs at framework region 1 (FR1) of the V_L_ region, with several contacts occurring outside of the V_L_ region: K107 between the V_L_ and C_L_ regions, E143 from the C_L_ region and R24 on the β-strand of CDR-L1 of IgM Fab. The interface includes residues from the α-helix and second β-strand of PpL domain B1 (122). The interface has a high affinity (K_d_: 110 nM) (123), forming a predominantly hydrophilic interaction characterised by 9 H-bonds, although several residues have been proposed as hotspots from *in silico* alanine scanning of the Fab and PpL (115, 123, 124). Interestingly, recent evidence showed distal FWR3 effects on the PpL binding site at the FW1 (125) adding to the considerations for the light chain pairing with the heavy chain (126, 127).

The second binding site is formed from 15 residues at β-strands A, B, C and D of the V_L_ region of IgM Fab, and the α-helix and third β-strand of PpL (**Figure 7D**). Although the second binding site is slightly larger and composed of more cross-interface contacts, it has a lower binding affinity (3.4 µM) (115). The first and second binding sites of PpL share only one common residue from PpL (R52) but 10 out of 15 residues from IgM Fab.

## B-Cell Superantigen-FC Binding

**Figure 8:**
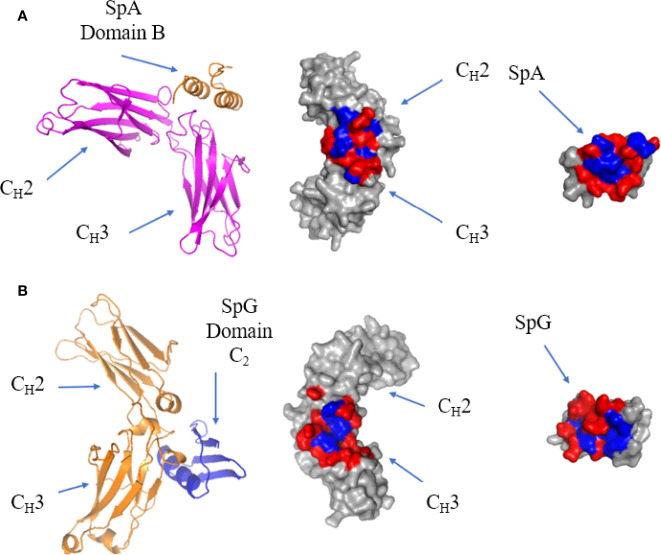
Binding of SpA and SpG superantigens to -IgG Fc. **(A)** Left panel: ribbon plot of SpA Domain B (Orange) bound to IgG Fc (Pink) at the C_H_2-C_H_3 interface (PDB code: 5U4Y). Middle panel: IgG Fc showing polar (red) and hydrophobic residues (blue). Right panel: SpA. **(B)** Left panel: ribbon plot of SpG Domain C2 (Blue) bound to IgG Fc (Orange) at the C_H_2-C_H_3 interface (PDB code: 1FCC). Middle panel: IgG Fc. Right panel: SpG.

The crystal structure of a single domain from SpA was determined in complex with IgG Fc: it showed that the protein-protein interface occurs between α-helix 1 and 2 of SpA domain B and C_H_2 and C_H_3 of the Fc (113) (**Figure 8A**). The residues forming the interface are generally hydrophilic (128). SpA residues Q9, Q10, D36 and D37, are conserved in the five immunoglobulin binding domains of SpA and are required for Fc binding (129). Mutating residue H435 in IgG eliminates SpA binding, as this residue is situated on the C-terminus of the C_H_3 region and protrudes into the C_H_2-C_H_3 cleft forming surface contacts with SpA (84).

The crystal structure of SpG C2 in complex with IgG Fc showed that it binds at the same site as SpA, with SpG binding to IgG Fc at the C_H_2-C_H_3 interface **(****Figure 8B**). SpG fits within the C_H_2-C_H_3 cleft and binds through residues on the α-helix and third β-strand. As they recognise essentially the same site, SpA and SpG bind competitively to IgG Fc (130, 131). The strong binding affinity of SpG for IgG Fc is contributed by a hydrophobic pocket surrounded by hydrophilic residues. Comparing the binding sites of SpG for Fab and Fc, Fab binding uses β-strands 1 and 2 as well as the α-helix, whereas Fc binding uses β-strand 3 as well as a more prominent contribution of α-helix residues.

## Physicochemical Characteristics of T and B Cell Superantigen Interfaces

**Figure 9:**
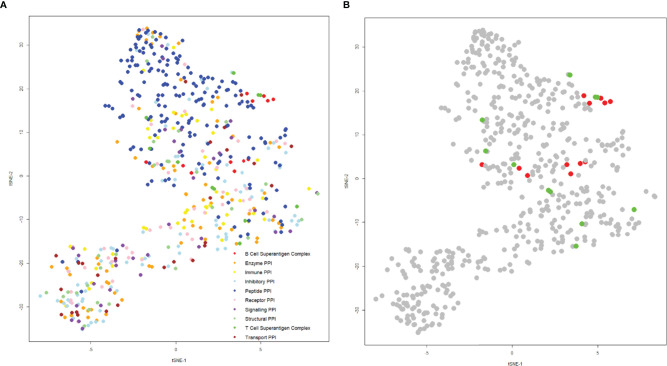
t-SNE plot of protein-protein interactions highlighting superantigen-antibody complexes. The position of each complex was determined using the following parameters: “Buried Surface Area (A^2^)”, “Buried Surface Area Hydrophobicity (A^2^)”, “Number of Interface Residues”, “H-Bonds”, “Salt Bridges”, Category”, “Total Positive Charge at Interface (A^2^)”, “Total Negative Charge at Interface (A^2^)” with a perplexity of 30. **(A)** Distribution of general protein-protein interactions categorized by function (T-cell superantigen complex, B-cell superantigen complex, enzyme PPI, immune PPI, inhibitory PPI, peptide PPI, receptor PPI, signaling PPI, structural PPI, toxin-antitoxin PPI and transport PPI). **(B)** The same plot as **(A)**, but coloured for T-cell superantigen complexes (green) and B-cell superantigen complexes (red).

The structures of the complexes of T and B-cell superantigens with immune macromolecules were examined to compare the nature of the interfaces with all other structures of protein-protein complexes. A list was compiled from the Protein Data Bank, extracting specific data on hydrophobicity, number of hydrogen bonds, salt bridges, interface area, binding affinity, and charges at the interface. These values were then condensed onto a two-dimensional plot using t-distributed stochastic neighbor embedding, such that each point represents a complex (**Figure 9**).

Complexes were categorized according to function: most were well dispersed by functional category, although peptide complexes tended to predominate in the upper half of the plot (**Figure 8A**). The superantigen complexes are grouped in the central and right side of the plot, indicating that their binding interfaces share some physicochemical characteristics (**Figure 9B**). An explanation for this phenomenon is that T-cell and B-cell superantigen interactions are transient-type complexes, as defined by Noreen and Thornton (132). Such complexes tend to be small and less hydrophobic than obligate, homo oligomeric complexes. Both T- and B-cell superantigen interfaces form interface areas less than 1000 Å^2^ and range from slightly to very hydrophilic.

The T-cell superantigens are located on the centre and right-hand side of the plot and are more scattered than the B-cell superantigen interfaces (**Figure 9B**). The interface areas of T-cell and B-cell superantigens have similar ranges: 436 – 944 Å^2^ and 517 – 714 Å^2^ respectively. Fractional hydrophobicity of the T-cell superantigen interfaces range from 18 – 49%, similar to those for B-cell superantigens (9 – 40%).

Although B-cell superantigens recognise different binding sites within the Fab molecule (V_H_, V_L_ and C_H_1) they share very similar interface physicochemical properties, which align closely to those seen in peptide complexes.

The fact that superantigens are promiscuous and capable of recognition of different binding partners indicates there is scope for improving binding affinity and extending specificity for specific targets. The observation that B-cell superantigens, and more specifically superantigen-Fab complexes, are physicochemically similar may allow for development of engineering strategies which makes use of this facility. Nonetheless, just as we expanded the definition of superantigens in this review to include B-cell activation based on new findings, we are also aware of novel superantigen-like behaviours by non-proteins e.g., nickel (133) that may in time be included as superantigens in the future.

## Superantigen Applications

Superantigens have been employed in multiple applications, both clinically and industrially. Though many improvements have been made, there is room to engineer and expand their scope and applications. Understanding the biochemistry of the superantigen-antibody interfaces provides an information resource for the development of novel biotechnological and pharmaceutical applications.

## Industrial

Since the approval of the first therapeutic monoclonal antibody in 1986 (Muromonab-CD3), the use of antibody-based drugs has expanded significantly with technological developments such as scFv, antibody-drug conjugates (ADCs) and bispecifics. In 2019, 70% of all biopharmaceutical products sold were monoclonal antibodies in a market worth over $150 billion. Antibody-based drugs continue to increase their market share, with current estimates predicting global revenue to increase to over $300 billion by 2025 (134). The expansion of antibody-based drugs has therefore created a need for improved manufacturing and purification processes.

The most prominent industrial application of B-cell superantigens is their use as affinity resins for the purification of antibodies, allowing highly efficient separation of antibodies for clinical and research applications. Improvement of these affinity resins has allowed pharmaceutical companies to develop cost-effective antibody purification techniques, increasing the feasibility of large-scale manufacturing of antibodies, resulting in the expansion of the industry. There are, however, some limitations; 80% of the downstream processing cost occurs at the capture and purification phase (135), and there is no single resin which can bind all antibody isotypes from all species of interest. Furthermore, antibodies are eluted from the affinity resins at low pH values, frequently causing aggregation.

Some investigators have engineered superantigens to optimize their application in antibody purification (136, 137). For example “Domain Z” was developed in 1987- a mutant of Domain B with two mutations, A1V and G29A (138), which resulted in SpA losing the ability to bind V_H_3-Fab while retaining Fc affinity (139); this innovation allows for the selective purification of the Fc fragment after pepsin digestion. One such Z domain affinity resin is Cytiva’s mAb Select SuRe (140). When testing mutant N23T, the stability of the SpA Z domain resin increased (141). Recently a new SpA resin has been developed: AviPure. This resin is formed of two B domains with two cysteine and histidine residues at the C-terminus to the steric hindrance, increasing binding capacity and increasing its resistance to pHs extremes, while retaining high binding affinity (135). Affinity resins undergo cleaning in place (CIP) procedures commonly using 0.5 M NaOH; therefore, affinity resins with high alkaline stability are desirable. To address the issue with CIP procedures, SpA was shown to have higher alkaline stability with a single mutation at position 29, with G29W being the most stable (142). Two further mutations N23T and F30A to the SpA Z domain resulted in a higher alkaline resistance when compared to wild type (143). SpA Z domain was also engineered to include six glycine residues on the second loop, which resulted in an increase in the elution pH (143). Wild type SpA is less susceptible to extreme alkaline conditions, with a half-life of 16 h (141) compared to SpG, which has a half-life of under 10 mins (144). Asn residues were identified as the most susceptible to deamination: mutation of all three Asn residues of SpG (N8T, N35A and N37A) improved alkaline stability by 8-fold (145). SpG was further demonstrated to have higher alkaline stability when three other mutations Y3F, T16I and T1I were introduced (145). By increasing alkaline stability, the lifespan of affinity resins can be increased, lowering the overall cost of antibody production. It has also been recently demonstrated that adding an additional alkaline wash step after the antibody capture step results in a decrease in antibody aggregation, lower impurity levels and an increase in antibody yield (146).

With the development of new formats such as single chain variable fragments (scFvs), strong Fab binding is required. Unfortunately, SpA and SpG have lower binding affinities for the Fab fragment compared to Fc. PpL has the advantage over other B-cell superantigens of binding strongly to the Vκ of scFv, (K_d_ = 4.5nM) (147). The scFv structural arrangement consists of the V_H_ and V_L_ domains connected by a short linker. scFv molecules have the advantage of retaining the CDRs while being significantly smaller than whole antibodies. SpA can also bind scFv but at a lower binding affinity than PpL, whereas SpG is unable to bind scFv. The most significant downside for the use of PpL as an affinity resin is its inability to bind λ light chains. Therefore, in human antibody production, roughly 34% of the antibodies will not bind to the resin, suggesting that engineering PpL to bind λ light chains could be valuable.

## Clinical Usage

### Diagnostics Potential

Superantigens are used to detect IgG in serum (148), making use of their immunoglobulin binding specificity. On the contrary, superantigens recognized by IgGs allow for the detection of *Staphylococcus aureus* (149, 150) in disease states.

Engineering of superantigens to be specific to regions of TCRs or antibody V-region families or isotypes for the development of diagnostic kits could be applied to the quantification of disease-associated proteins e.g. IgE in allergy. The ability to specifically bind antibodies can also allow its development in easy-to-use, non-technical point-of-care testing home-use devices (151), as recently applied during the COVID-19 pandemic. Such superantigen-based diagnostics can be coupled with colorimetric, home-made devices [e.g. mobile spectrophotometers (152, 153)]. Given the increasing association of antibody V_H_ families with certain diseases e.g. [V_H_5 in nickel allergy (85)], superantigens that can differentiate antibody V_H_ families have clear potential in diagnostic kit development.

### Therapeutic Potential

The role of superantigens in sepsis, a leading cause of death listed by the WHO, makes them an important target for toxic-shock syndrome (154). Several short peptide regions (~40 residues) from SEA and SPEA have been identified as causes of vasodilation (155), suggesting an application in the development of antihypertension drugs.

Superantigens can also be used as a target for an anti-*Staphylococcus aureus* vaccine. There have been several attempts at producing a vaccine against *S. aureus*, without success, although it has been shown that the use of anti-SpA antibodies leads to the promotion of opsonophagocytic clearance of *Staphylococcus aureus* (156, 157).

Superantigens have also shown promise in the treatment of cancer through a synergistic effect with antibodies in the recruitment of T-cells (158). The ability of SEB to hyper-stimulate and proliferate CAR T-cells led to a more effective antitumour response when used in combination (159). PpL has been shown to induce apoptosis in malignant κ^+^ B cell lymphomas in humans and mice (160), demonstrating the potential use of superantigens as anti-cancer drugs, particularly when sagaciously paired with a suitable V_κ_ light chain (94). A range of potential T-cell superantigen-based anticancer drugs have been recently reviewed (161), including SEB, demonstrating the ability to inhibit metastasis and tumour growth (162). Several Fab-superantigen fusion proteins show promising Phase I/II clinical trial results. A major drawback with using superantigens is their potential to elicit a toxic response. One way to prevent this is to reduce the over-stimulation of T-cells. SEA was split into two functionally inactive domains and attached to a scFv. When used in combination, the two SEA fragments reassemble, forming a functionally active superantigen and resulting in the selective activation of T-cells (163). Another way to avoid superantigen toxicity is to utilize superantigen-like proteins which, as mentioned previously, are very similar in structure and function, although they do not result in emesis. They have been shown to inhibit tumour growth by 30% without significant toxicity (164).

The importance of understanding superantigens goes beyond bacterial sepsis to viruses, where for example, SARS-CoV2 spike protein displays superantigen properties (165, 166) causing multisystem inflammatory syndrome in children through its unspecific activation of T-cells (167).

## Summary

With the development of new clinical therapeutics, B-cell superantigen engineering presents an opportunity to develop novel applications, as well as improving current superantigen-based technology, such as purification resins. Structural information on B-cell superantigen interfaces has been useful in providing a basis for the engineering of binding characteristics. The application of protein engineering principles offers considerable scope for directed modification of superantigen binding properties and harnessing for applications in medicine and the pharmaceutical industry.

## Author Contributions

AD wrote the manuscript, with critical revisions from SG and JD. AD and JD prepared the figures. All authors contributed to the article and approved the submitted version.

## Funding

AD is a PhD student funded by The University of Manchester, UK and the Agency for Science, Technology and Research (A*STAR), Singapore. This review was partially funded by the National Research Foundation (NRF) Singapore grant to Experimental Drug Development Centre (EDDC), ASTAR.

## Conflict of Interest

The authors declare that the research was conducted in the absence of any commercial or financial relationships that could be construed as a potential conflict of interest.

## Publisher’s Note

All claims expressed in this article are solely those of the authors and do not necessarily represent those of their affiliated organizations, or those of the publisher, the editors and the reviewers. Any product that may be evaluated in this article, or claim that may be made by its manufacturer, is not guaranteed or endorsed by the publisher.
